# Changes in the incidence of stress reactions and fractures among intercollegiate athletes after the COVID-19 pandemic

**DOI:** 10.1186/s13018-023-04282-7

**Published:** 2023-10-20

**Authors:** Brendan Y. Shi, Chloe Castaneda, Varun Sriram, Stefani Yamasaki, Shannon Y. Wu, Thomas J. Kremen

**Affiliations:** 1 Department of Orthopaedic Surgery, David Geffen School of Medicine at University of California, Los Angeles, Los Angeles, CA USA; 2 1225 15th Street, Suite 2100, Santa Monica, CA 90404 USA

## Abstract

**Purpose:**

The purpose of this study was to characterize the impact of detraining due to the COVID-19 pandemic on incidence of bony injuries and stress fractures in collegiate athletes.

**Methods:**

A comprehensive collegiate athletic conference injury database was queried for all in-season, sport-related bony injuries (defined as all stress reactions and fractures) that occurred across all sports from January 2016 to June 2021. The bony injury rate per 1000 athlete exposure hours (AEH) was calculated and compared between the immediate post-hiatus season and historic rates from pre-hiatus seasons (2016–2019). Injury characteristics were also compared between the pre- and post-hiatus time periods.

**Results:**

A total of 868 bony injuries across 23 sports were identified. The sports with highest overall baseline bony injury rates in historic seasons were women’s cross country (0.57 injuries per 1000 AEH) and men’s cross country (0.32). Compared to historic pre-hiatus rates, female cross-country runners demonstrated a significantly lower bony injury incidence rate in the post-hiatus season (0.24 vs. 0.57, *p* = 0.016) while male swimming athletes demonstrated a statistically significant increase in bony injury rate (0.09 vs. 0.01, *p* = 0.015). The proportion of bony injuries attributed to repetitive trauma increased; while, the proportion of injuries attributed to running decreased between the pre- and post-hiatus seasons.

**Conclusion:**

Across all sports, there was no consistent trend toward increased rates of bony injury in the immediate post-hiatus season. However, female cross-country runners demonstrated lower rates of bony injury in the post-hiatus season while male swimmers demonstrated higher rates. Furthermore, bony injuries in the post-hiatus season were more likely to be the result of repetitive trauma and less likely to be from running.

**Level of Evidence:** Level III, retrospective, cross sectional study.

## Introduction

Coronavirus disease 2019 (COVID-19) caused a worldwide pandemic with over 140 million confirmed cases and nearly 3 million deaths [1, 2]. In response, strict public health measures including social distancing and cancelation of organized group activities were enacted to decrease transmission of disease [3]. One of the activities that experienced significant interruption during the implementation of quarantining was organized sports. In the Pacific 12 conference (Pac-12) of the NCAA, student athletes were unable to compete with their team in any official capacity from March 2020 to November 2020. Some teams experienced an even greater delay to resuming competitions and organized practices. During this period, athletes were unable to access the facilities, staff, and coaching provided by their university. Furthermore, they were removed from the regular training routine and specialized resources that were designed to prevent general and sport-specific injuries.

One of the potential consequences of this abrupt pause in their training is an increase in injury risk, especially stress-related injuries to bone, after resuming athletic activities. When determining proper recommendations to prevent stress fractures among athletes, Bennell et. al. demonstrated that gradual, stepwise escalations in activity are beneficial during a period of training increase [4]. In contrast, improper workload management, especially at the beginning of the season, can disrupt bone remodeling and increase risk for stress injuries to bone [5, 6]. Certain weight bearing areas of bone are incredibly sensitive to changes in physical activity and, as a result, pauses in regular activity can result in significant loss of bone mineral density [7].

In light of this, athletes that are re-introduced to high physical workloads after being away from normal training resources for at least 8 months may be at an elevated risk of bony stress injuries upon resumption of play, particularly if a stepwise increase in activity was not conducted. Multiple prior studies have shown that athletes are at increased injury risk after long pauses in athletic activity [8–10]. After the 14-week lockout prior to the 2011 National Football League season, players returning to play suffered significantly increased rates of Achilles tendon rupture [10]. More recently, during the COVID-19 related stoppages of various professional sports leagues, rapid return to normal competition resulted in significant increases in certain injuries compared to pre-COVID-19 seasons. Major League Baseball saw a significant increase in upper extremity injuries and foot/ankle injuries during the 2020 season compared to the 2018–2019 seasons [9]. The National Football League also observed an increase in injury rate, likely due to an increase in the amount of time spent training and intensity of competition, compounded further by detraining associated with the pandemic [8]. These findings were consistent in the German professional soccer league as well [11].

While the effects of detraining due to the COVID-19 pandemic have been explored in professional athletes, its impact on bony stress injuries in collegiate athletes has not been characterized. Furthermore, prior studies have not assessed the effect of long periods of unsupervised training on sports such as swimming, cross country, gymnastics, or track and field. Using the comprehensive Pac-12 Health Analytics Program (HAP) database, this study aimed to compare the rates of stress fractures and stress reactions of bone among Pac-12 athletes before and after the COVID-19 pandemic associated hiatus of intercollegiate athletic activities, (hereafter refer to as “hiatus”). We hypothesized that athletes in the post-hiatus time period would demonstrate higher rates of bony injuries and stress fractures when compared to historic rates.

## Methods

### Database

The Pacific 12 (Pac-12) Health Analytics Program (HAP) Sports Injury Research Archive is an externally validated, conference-wide database that contains data on all injuries that take place during official NCAA competitions or practice among intercollegiate Pac-12 athletes. Injury data are submitted by athletic trainers and health care providers that have been specifically trained in the standardized data collection workflow. The submitted data are then reviewed by designated campus administrators (either certified athletic trainers or health care providers) who also oversee data entry and implement weekly quality checks. Finally, the data are de-identified and moved to the data repository. This database has been externally reviewed and validated by a third party data analytics firm [12].

A data request was submitted to the HAP for all in-season, sport-related bony injuries that occurred across all sports from January 2016 to June 2021. Injuries designated as “stress reaction,” “stress fracture,” “fracture,” or “bone stress injury” were included. After the data use agreement was processed between the primary institution and the Pac-12 conference, we received a fully de-identified data file containing the data elements of interest via a secure cloud-based file sharing system. This study was exempted from IRB review due to utilizing fully de-identified data.

### Data elements

Collected demographic data elements included gender, sport, and calendar year of injury occurrence. Injury specific data elements available within the database included injury etiology, timing of onset, severity (season ending or able to return to competition), procedural intervention, injury mechanism (contact, acceleration/deceleration, jumping, repetitive trauma, running, sprinting, throwing), and event segment that injury took place. Within the HAP database, all sporting events (practices or games) are divided into quartiles, and the quartile in which the injury took place was recorded for each injury. This was performed even for events or sports (such as soccer or baseball) that do not have traditional “quarters” of play. The HAP database defines timing of injury onset as acute if the athlete presented within 24 h of injury event and as overuse for all presentations more than 24 h after injury event.

Injuries were categorized as pre-hiatus or post-hiatus based on calendar year of injury and each sport-specific schedule. For example, injuries occurring in 2020 were categorized as post-hiatus for certain fall sports (e.g., football) since football largely resumed competition by October 2020. Since spring sports such as baseball resumed competition later, baseball injuries occurring in 2021 were categorized as post-hiatus.

Athlete bony injury incidence rates (IR) per 1000 athlete exposure hours (AEH) were determined by taking the number of total in-season injuries and dividing by total athlete time at risk. Similar to prior literature [13, 14], injury rate was calculated using the following equation:

IR per 1000 AEH = [(Number of total in-season injuries)/(Season length in weeks * 20 h/week * number of athletes on the team)] * 1000.

To estimate time at risk, total season length in weeks was multiplied by 20 h per week as this is the maximum duration of sport-related activity according to NCAA bylaws. Team rosters as well as season start and end dates were obtained from publicly available schedules on the Pac-12 website (www.pac-12.com).

### Statistical analysis

All recorded injury specific data elements were compared between the immediate post-hiatus season (either 2020 or 2021, depending on sport) and historic rates from pre-hiatus seasons (2016–2019). Sub-group analyses were performed by sex and specific sport.

Bony injury incidence rate ratios per sport were calculated between the incidence rate in post-CASIA seasons and the historic incidence rate in pre-hiatus seasons. Pearson’s chi-squared test was used to analyze all categorical variables. Alpha level was set at 0.05 for all statistical tests. All data were analyzed using Stata 12 software (Statacorp LLC, College Station, TX).

## Results

A total of 868 bony injuries across 23 sports were identified, with 699 in the 4 pre-hiatus seasons pre-hiatus and 169 in the post-hiatus season.

The sports with highest overall baseline bony injury rates in pre-hiatus seasons were women’s cross country (0.57 injuries per 1000 AEH), men’s cross country (0.32), women’s basketball (0.20), women’s gymnastics (0.16), women’s soccer (0.13), and men’s volleyball and basketball (0.13 each) (Table 1).

**Table 1:** Bony injuries per 1000 athlete exposure hours in largest NCAA sports

Sport	Pre-Hiatus Injury rate	Post-Hiatus injury rate	*p* value
Women’s basketball	0.20	0.11	0.333
Women’s gymnastics	0.16	0.22	0.505
Women’s rowing	0.07	0.07	0.881
Women’s soccer	0.13	0.11	0.695
Women’s softball	0.06	0.10	0.249
Women’s swimming	0.01	0.00	n/a
Women’s tennis	0.08	0.07	0.803
Women’s track	0.11	0.12	0.946
Women’s volleyball	0.11	0.08	0.528
**Women’s cross country**	**0.57**	**0.24**	**0.016**
Men’s baseball	0.05	0.09	0.055
Men’s basketball	0.13	0.14	0.851
Men’s football	0.10	0.10	0.789
Men’s gymnastics	0.03	0.00	n/a
Men’s rowing	0.03	0.01	0.226
Men’s soccer	0.11	0.22	0.105
**Men’s swimming**	**0.01**	**0.09**	**0.015**
Men’s tennis	0.06	0.03	0.594
Men’s track	0.07	0.08	0.550
Men’s volleyball	0.13	0.06	0.530
Men’s cross country	0.32	0.20	0.302

Sports with statistically significant differences between pre- and post- hiatus injury rates are bolded

**Fig. 1:**
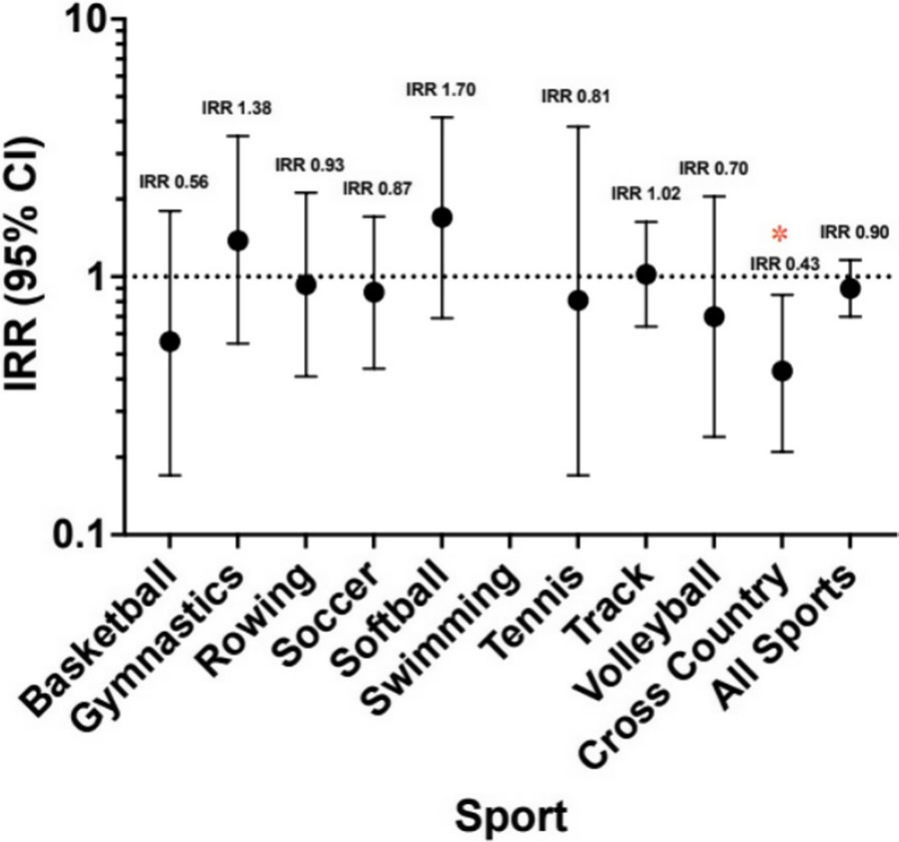
Female Pac-12 athlete bony injury incidence rate ratio (IRR). Value > 1 indicates higher rate post-hiatus

**Fig. 2:**
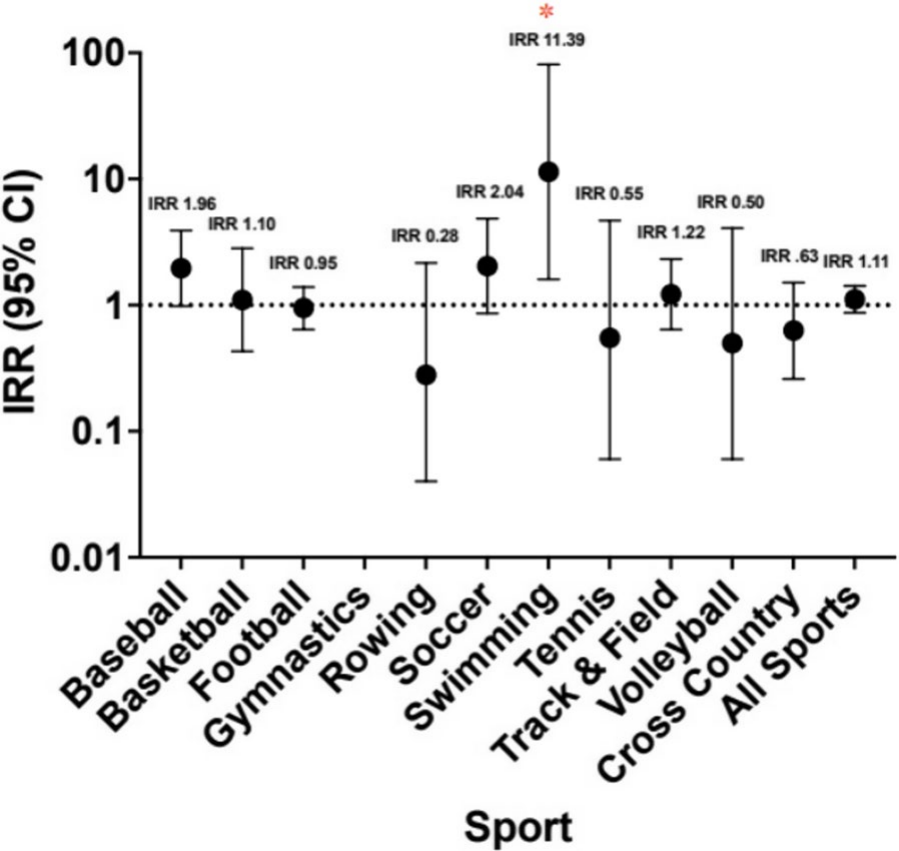
Male Pac-12 athlete bony injury incidence rate ratio (IRR). Value > 1 indicates higher rate post-hiatus

For the majority of sports, there was no significant difference in bony injury incidence rate between the pre- and post-hiatus time periods. However, female cross-country runners, the athlete demographic with the highest historic rate of bony injury pre-hiatus, demonstrated a significantly lower bony injury incidence rate of 0.24 injuries per 1000 AEH in the post-hiatus season (Incidence Rate Ratio (IRR) 0.43, 95% confidence interval 0.21–0.85, *p* = 0.016) (Fig. 1). There were a total of 72 bony injuries in female cross-country runners in the pre-hiatus seasons, compared to 9 injuries in the post-hiatus season. In men’s swimming, there were 2 total bony injuries in both the pre-hiatus seasons and the post-hiatus seasons. However, due to a tenfold decrease in exposure hours, there was a statistically significant increase in the rate of bony injury from 0.01 to 0.09 injuries per 1000 AEH between the pre- and post-hiatus time periods (*p* = 0.015) (Fig. 2).

Across all sports, the proportion of bony injuries attributed to a repetitive trauma mechanism increased from 8.7% pre-hiatus to 16.6% post-hiatus (*p* < 0.001) while the proportion of these injuries attributed to a running mechanism decreased from 25.2% pre-hiatus to 17.2% post-hiatus (*p* < 0.001). There was no difference between pre-hiatus and post-hiatus time periods with respect to proportion of female sex, rate of procedural intervention, timing of onset, rate of season ending injury, rate of 4^th^ quarter injury, or rate of new versus recurrent bony injury (Table 2).

**Table 2:** Pre- and post-hiatus bony injury characteristics in Pac-12 division 1 NCAA intercollegiate athletes

	Pre-hiatus (%)	Post-hiatus (%)	*p* value
Female sex	51.1	46.8	0.313
Procedural intervention	1.4	1.8	0.741
Overuse injury	50.4	46.8	0.399
Season ending	3.4	2.4	0.481
4^th^ quarter injury	29.9	20.0	0.054
New injury	89.4	89.4	0.990
Contact mechanism	42.2	41.4	0.425
Repetitive trauma mechanism	**8.7**	**16.6**	** < 0.001**
Running mechanism	**25.2**	**17.2**	** < 0.001**

Sports with statistically significant differences between pre- and post- hiatus injury rates are bolded

## Discussion

In this comprehensive study of bony injuries across the Pac-12, we found no overall significant increase in injury incidence rate in the post-hiatus season when compared to historic averages. However, we were able to uncover differences in bony injury rates in specific sports. While the injury rate in men’s swimming was higher in the post-hiatus season, the injury rate in women’s cross country was lower. Furthermore, in the post-hiatus season, overall, a higher proportion of bony injuries was noted to be the result of repetitive trauma and a lower proportion was attributed to running. We found no significant differences in the proportion of acute bony injuries, the proportion of injuries sustained in the final quarter of competition or practice, the proportion of non-contact injuries, or the proportion of season ending injuries between pre- and post-hiatus seasons.

While male and female cross-country runners had the highest overall bony injury incidence rate of all athletes in this study, we found that the injury rate in female cross-country athletes was actually lower in the post-hiatus season compared to pre-hiatus. It is possible that an initial break from running followed by a gradual increase in running during the pandemic was beneficial. Historically, the rate of injury in runners has been well characterized—most are overuse injuries and women are at a higher risk than men [15–17]. Bennell reported a 21% incidence of stress fractures in track and field athletes, with risk factors including low bone mineral density, menstrual disturbance, low lean mass in lower limbs, leg length discrepancy, and a low fat diet [18]. For both men and women, stress fractures made up the highest proportion of “severe injuries” [16]. Scofield et al. noted the increased risk that endurance athletes have in developing bony injuries due to their relative decreased bone mineral density when compared to other athletes, and the repetitive impact that is inherent to certain endurance sports such as longer distance running [15]. The repetitive impact experienced by the lower extremities of runners results in micro-injuries within bone and subsequent bone remodeling [19]. The remodeling period can take up to 3–4 months in cortical bone and further micro-injuries during this period can compound the damage and lead to stress fractures [19]. It is possible that the initial quarantine period was a welcome pause for certain female runners, allowing adequate bone remodeling to occur away from the rigor of daily organized practices. Finally, it is likely that runners were able to run on their own to maintain conditioning even during the official pause in athletic activity. Resumption of running-based training in the outdoor environment (even among institutions with traditionally indoor facilities) was likely beneficial to cross-country runners, providing more ideal physiologic cues to optimize the bone strength and bone mineral density necessary for running prior to entering the post-hiatus season.

Conversely, swimmers were among the least likely athletes to be able to engage in sport-specific activities during the hiatus, as it is probable that finding a pool suitable for high intensity training particularly with multiple teammates was difficult to impossible in most cases. These athletes’ increased rate of recorded bony injury upon returning to organized on-campus training in the post-hiatus season may reflect their dependence on higher impact training modalities (such as running) in the absence of pools. Finally, the overall low number of bony injuries in swimming makes it susceptible to spurious findings. With only 2 bony injuries among male swimmers during both the pre-hiatus and post-hiatus time periods, it is likely that the statistically significant difference may not have clinical relevance.

Overall, across all sports and all athletes there was no significant difference in the bony injury incidence rate between the pre- and post-hiatus time periods. This may be a surprising result given prior studies in professional leagues demonstrating increased injury rates after a hiatus in competition. However, there are several potential reasons for this, including the delayed start to the post-hiatus seasons in the Pac-12 and the relatively younger age of collegiate athletes. While official athletic activity was allowed to resume in November 2020 for all Pac-12 sports, many sports such as soccer, swimming, cross country, and field hockey, which traditionally began their seasons in the fall, were delayed to 2021, allowing for a period of time to ramp-up athletic activities prior to the beginning of competitions. This delay in start of competition may have allowed athletes to return to their pre-hiatus level of fitness, attenuating the endurance loss and acute versus chronic workload mismatch that could put them at higher risk of sustaining an injury. It is possible that Pac-12 athletes were afforded enough time for a gradual increase in workload during the return to sport phase such that they could avoid bony/stress injuries often attributed to a decrease in endurance. Furthermore, for many sports the post-hiatus “in season” competition schedules were shortened to have significantly fewer competitions compared to pre-hiatus seasons. It is possible that the truncated post-hiatus seasons may not have been long enough for the previously experienced period of detraining to have detectable significant detrimental effects on bone health.

Athlete age may have also contributed to the lack of difference found in bony injury rate in our study. Age has been hypothesized to be a notable variable in the incidence of bony stress injuries among athletes due to alteration in bone architecture among other factors [20]. While there is a lack of definitive evidence associating higher age with a decreased bone mineral density, Mattila et al. found older age to be a significant risk factor for bony stress injuries in military recruits [21]. Furthermore, Rizzone et al. demonstrated that 22% of stress fractures suffered over a 10-year period among NCAA athletes were recurrent injuries [22]. Compared to professional athletes, collegiate athletes are generally younger and less likely to carry previous injuries. Overall, the lower likelihood of prior injury, delayed start to competition, and biological factors associated with age [23] potentially contributed to the lack of difference in bony injury rate observed between collegiate athletes before and after the COVID-19 hiatus in our study.

Regarding the mechanism of injury after return to sports, a higher proportion of bony injuries was due to repetitive trauma and a lower proportion was due to running in the post-hiatus season. While we hypothesized that running would comprise a higher proportion of bony injuries post-hiatus compared to pre-hiatus, it is possible that the ability of athletes to run outside during the COVID-19-associated quarantine period allowed them to maintain their running-specific fitness. Hence, post-hiatus running likely put similar amounts and types of stress on their bones, allowing for the incremental increase in physical load that Bennell et al. postulated would decrease injury incidence, specifically as it pertains to running [4]. In short, the detraining period for running was likely attenuated.

The lack of significant differences between pre- and post-hiatus with respect to injury onset, recurrence, timing, and mechanism may be due to risk factors not captured in the HAP database such as experience in the sport, vitamin D status, nutritional factors or history of stress injuries. Furthermore, physical characteristics that influence bone health that are expected to differ between sports might have had an influence on bony stress injuries, including the general height and weight of an athlete [15]. The attributes that help athletes be successful in their respective sports produce an incredibly diverse population of Pac-12 athletes with respect to physical build. It is important to give careful consideration to the different factors that affect an athlete’s likelihood to suffer bony injuries, and to ensure that measures are taken to produce comprehensive protocols for injury prevention and management using the best available clinical evidence.

## Limitations

Although this analysis utilizes a large database involving comprehensive injury data provided by the Pac-12, there are several major limitations. The primary limitations are due to characteristics inherent to the HAP database, many of which fail to adhere to the International Olympic Committee (IOC) consensus statement from 2020 [24]. First, the HAP database does not distinguish between injuries that occur during a game or practice, despite evidence that injury rates are different during competition versus training. Second, the HAP database does not report the type of personnel (athletic trainer versus healthcare provider) that initially reported the injury. Although the HAP database undergoes multiple rounds of rigorous quality checks^12^, the heterogenous nature of data collection may affect the accuracy and appropriate classification of injury-related information. Third, the classification of the recurrence of an old injury versus the formation of a new injury is subject to imprecision based on what an athlete believes constitutes a prior injury. Fourth, the HAP database only reports the year that the injury event takes place, rendering it impossible to comment on the effect of fatigue toward the end of the season. Fifth, the HAP database defines all injuries as acute if the athlete presents within 24 h of the injury event and “overuse” for presentations past 24 h. This classification is flawed and should be more aptly characterized as non-acute. Finally, detailed information such as athlete age, level of training during the pandemic, vitamin D status, and other important factors that likely have an influence on the rate of bone stress injuries are not captured in the HAP database in an effort to protect athlete identity in accordance with our data use agreement. Despite the limitations of the HAP database, the collection and coding process stayed consistent during the duration of the study period. Hence, it is reasonable to also assume that any systemic issues in data collection that occurred pre-hiatus would also be seen post-hiatus. Given this, we believe that discrepancies in our methodology from the IOC consensus do not influence our primary finding that there were similar bony injury rates pre- and post-hiatus in the majority of NCAA sports.

There are additional limitations to the study. Athlete exposure hours were estimated based on NCAA bylaws restricting student-athletes to 20 h per week. It is not known what the actual training hours were for each athlete, nor is it known whether there were additional restrictions with respect to training time or participation intensity after the pandemic.

## Conclusion

Across all sports, there was no consistent trend toward an increased rate of bony injury in the immediate post-hiatus season. Female cross-country runners demonstrated a lower rate of bony injury in the post-hiatus season and male swimmers demonstrated a higher rate. Bony injuries in the post-hiatus season were more likely to be the result of repetitive trauma and less likely to be related to running. Overall, these findings demonstrate that the pandemic most likely did not have a major effect on the rate of bony injuries in college athletes. As the hiatus had a variable impact on athletes from different sports, the importance of implementing return to sport protocols that aim to address the sport-specific effects of detraining should be emphasized.

## Data Availability

All data used for this study is available upon reasonable request.
